# Relay Selection for Capacity Increase in Underwater Acoustic Sensor Network

**DOI:** 10.3390/s21196605

**Published:** 2021-10-03

**Authors:** Ramsha Narmeen, Jaehak Chung

**Affiliations:** Department of Electronics Engineering, INHA University, Inhceon 22201, Korea; 22172307@inha.ac.kr

**Keywords:** underwater acoustic sensor network, relay node, propagation delay, cooperative communication

## Abstract

In long distance sensor nodes, propagation delay is the most crucial factor for the successful transmission of data packets in underwater acoustic sensors networks (UWAs). Therefore, to cope with the problem of propagation delay, we propose examining and selecting the best relay node (EBRN) technique based on checking the eligibility and compatibility of RN and selecting the best RN for UWAs. In the EBRN technique, the source node (S) creates a list of the best RNs, based on the minimum propagation delay to the midpoint of a direct link between S and the destination node (D). After that, the S attaches the list of selected RNs and transmit to the D along with data packets. Finally, from the list of selected RNs, the process of retransmission is performed. To avoid collision among control packets, we use a backoff timer that is calculated from the received signal strength indicator (RSSI), propagation delay and transmission time, whereas the collision among data packets is avoided by involving single RN in a particular time. The performance of the proposed EBRN technique is analyzed and evaluated based on throughput, packet loss rate (LR), packet delivery ratio (PDR), energy efficiency, and latency. The simulation results validate the effectiveness of the proposed EBRN technique. Compared with the existing schemes such as underwater cooperative medium access control (UCMAC) and shortest path first (SPF), the proposed EBRN technique performs remarkably well by increasing the throughput, PDR, and energy efficiency while decreasing the latency and LR in UWAs.

## 1. Introduction

More than 70% of the earth’s surface is covered with water. Numerous underwater acoustic (UWA) communication methods have already been developed to monitor the ocean environments [1,2,3,4]. Underwater sensors plays a vital role in collecting the environmental information and transmitting the gathered data to the buoy that transfers all the collected data to the surface station. The UWA communication signals suffer from a short communication range and a low bit error rates (BER) performance due to long propagation delays in time-varying UWA channels [5]. Acoustic propagation is characterized by three major factors, i.e., attenuation that increases with signal frequency, time-varying multipath propagation and noise [6]. The background noise is not white noise, although it is often characterized as Gaussian and it has a decaying power spectral density. In addition, severe multipath fading and large path loss cause a high BER of the communication links [7,8]. Compared to radio frequency (RF) signals, the UWA signal suffers from severe multipath fading, high BER, long propagation delays, and high attenuation in the channel [9]. The transmission loss in an underwater acoustic network not only depends on the signal frequency but also the distance between transmitter and receiver [10,11,12,13]. The motion of waves causes an extreme doppler effect [14], and the delay spread over hundreds of milliseconds results in frequency-selective signal distortion. Due to long propagation delay, the packet collision frequently occurs in the underwater acoustic communication system [15,16,17]. In UWA communication, the time-varying propagation, i.e., the speed of sound of 1500 m/s, causes the long delay between two communication nodes and results in the long delay channel profile [18]. In time-varying UWA channel, cooperative communication [19,20,21,22] is a promising technique. In cooperative communication, the participating nodes are source node (S), destination node (D), and relay node (RN). The RN, i.e., the cooperators, provides alternative paths between S and D to cope high BER.

### 1.1. Related Work

Many cooperative communication protocols for the radio frequency (RF) wireless networks have been developed [23], and the carrier sense multiple access with collision avoidance (CSMA/CA) is utilized for random access protocol [24]. Due to limited energy of UWAs, sensors cannot continuously sense the environment as in CSMA/CA. For this reason a cooperative communications is utilized in [25]. In this paper, the selection of the best RN is necessary to increase the communication link performance. In general, the RN is selected using the channel state information (CSI) among nodes. The estimation of the exact CSI between the neighbor nodes is difficult in the UWA channels. In Cooperative diversity multiple access and collision avoidance (CD-MACA) proposed in [25], all the RNs overhear the communication and store the data in a buffer to send it to the desired D. In [26,27], the authors proposed a distributed RN selection scheme with no global topology. Authors used opportunistic relaying (OR) in which each RN overhear request to send (RTS)/clear to send (CTS), deduce the channel quality, and derive a timeout time. Time-out time is a back-off time which is utilized by the RN for sending the overheard data to D. By utilizing OR, all RNs transmits packets at the same time that enormously increases the number of collision. In [28], UCMAC scheme selects the single best RN limiting the collision, for the process of retransmission. In this scheme, the D must select its nearest RN for the retransmission process. Authors in [28] selects the nearest RN is the one who has the better channel and minimum propagation delay from the D. At first, the D asks for the retransmission to the nearest RN, then the 2nd nearest RN, and so on. However, S to RN link quality is not delectable because of large distance between them and RN is unable to save the data packet in a buffer, thus it is incapable to transfer it to D. In [29], the authors proposed the shortest path first (SPF) scheme, in which the RN sends the RN-D signal-to-noise ratio (SNR) information by overhearing CTS sent by the D. S averages the two SNR values, arranges the list of cooperating RNs, and selects the RN with the minimum time-of-arrival (ToA). Generally, RN with minimum ToA is actually lied nearest to S and furthest from D causing bad link quality of RN-D.

### 1.2. Motivation and Objectives

For the reliable transmission of the data packet, we utilize a cooperative 4-way handshaking mechanism (RTS/CTS/Data/ACK) such that the success/failure of transmission/reception of control and data packets is detected intermittently. Due to time-varying propagation of signal, collision among control packets is occurred which is efficiently handled by using backoff timer. Additionally, the system must select the single optimal RN for the process of retransmission to limit the collision of data packets. The main contribution of this paper is summarized below

- The list of selected RNs is created by summing the two link propagation delays (S-RN and RN-D). RNs are arranged in an order that the top most RN has the minimum distance to the midpoint of direct link (S-D). The arranged list of selected RNs is attached along with data packet.
- Each RN, whose name is available in the list of best RNs, buffer the data packet while remaining RNs discards the received data packet.
- When D receives erroneous data packet, it asks for retransmission by broadcasting NACK until the data packet is received successfully.

The rest of the paper is organized as follows. In Section 2, we present the UWA system model and UWA channel characteristics. In Section 3, we present the proposed examining and selecting the best RN scheme. In Section 4, the performances of the proposed scheme are evaluated by computer simulations. Finally, Section 5 concludes this paper.

## 2. UWA System Model

**Figure 1:**
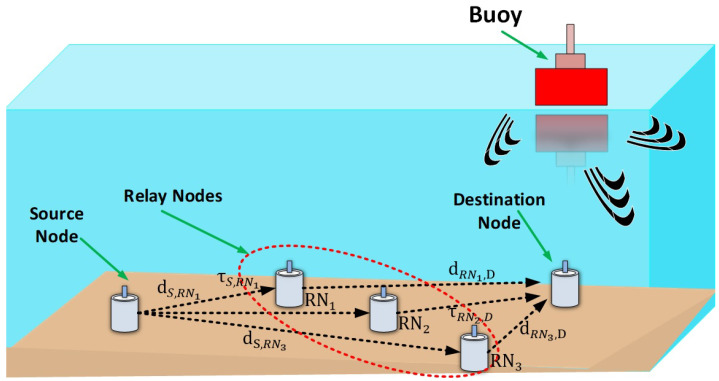
Network layout of cooperative communication for UWA.

The UWA sensor network consists of source node, destination node, and relay nodes, as shown in Figure 1.

Here, $d_{S,RN_{i}}$ and $d_{RN_{i},D}$ denote the distances from S to RN and from RN to D, respectively and *i* is the total cooperating nodes. $\tau_{S,RN}$ and $\tau_{RN,D}$ denote the propagation delays from S to RN, and RN to D, respectively. The, RN1, RN2, RN3 show the cooperating RNs. Here, S sends the gathered data to the D that transferred it to the surface buoy. When the distance between S and D is very large, direct communication from S to D will definitely cause failure. The probability for the first-time failure of the direct link between S and D is given as

(1) $$Pr\left(\gamma_{S,D}<\beta_{2\alpha }\right)=Pr\left(FC_{S,D}>\frac{\beta_{2\alpha }Z}{TP}d_{S,D}^{\zeta }\right).$$

where $\gamma_{S,D}$ denotes the received SNR of the direct link between S and D, $\beta_{2\alpha }$ denotes the minimum required threshold for the received packet which ensures the quality of packet, and $FC_{S,D}$ denotes the fading coefficient that is independent for the nodes S and D, $d_{S,D}^{\zeta }$ is the total Euclidean distance between S and D, *Z* and TP denote the noise power and transmission power, respectively. Equation (1) shows that the quality of received signal decays rapidly after passing through the channel.

## 3. Proposed Examining and Selecting the Best Relay Node (EBRN) Scheme

In Examining and Selecting the Best Relay Node (EBRN) scheme, the list of best RNs is developed, based on minimum distance to the midpoint of direct S-D link. In this way, the two links, i.e., S-RN and RN-D are the most reliable than any other possible path. Such that, when RN nearest to S, it would be furthermost to D, possibly worst RN-D link (SPF). Similarly, when RN is nearest to D, the overall path is worst due to bad S-RN link (UCMAC). Hence, we selected RNs which are nearest to midpoint to balance the quality of two links and calculated the best possible alternative path. Moreover, we utilize collision avoidance technique by considering received signal strength indicator (RSSI) when a control packet is sent from RN to S, propagation delay from S to RN and transmission time of control packet. The proposed EBRN scheme consists of two phases: The first is an initial channel reservation phase, and the second is a data transmission phase. In the initial channel reservation phase, RNs checks whether RNs lie between S and D or not. If RNs are located between S and D, they respond with the control packet to S. S selects and creates the RN candidate list for the retransmission. In the data transmission phase, S transmits the data packet to D. If D detects error in the data packet, it broadcasts NACK, and the first priority RN retransmits the data packet to D, and if D receives the erroneous data packet again, it broadcasts NACK once again and the next priority RN retransmits the data packet to D. The following are the assumptions of the proposed EBRN scheme:

- All nodes have the same transmission power and receiver capability.
- Every node is fixed on a seabed and is unable to acquire a timing synchronization due to the UWA channel characteristics.
- All nodes know the distance and the propagation delay time from/to one-hop neighboring nodes through the network initialization.
- The reactive type of the decode-forward (DF) signaling strategy is utilized on each RN.
- The errors occur only in data packets, not in the control packets.

Our proposed technique is effectively resolving the problem of time-varying characteristics of UWA channel such as collision due to random delay, high energy consumption, wastage of energy, and high BER. The reliable transmission of the data packet is achieved by taking advantage of cooperative communication/relaying somewhere between long range transmissions. In cooperative communication, the participating nodes are source node (S), destination node (D), and relay node (RN). The RN, i.e., the cooperators, provide alternative paths between S and D to cope high BER.

### 3.1. Initial Channel Reservation Phase

#### 3.1.1. Estimate RN Eligibility and Compatibility

Each RN first check its eligibility and then estimates the compatibility before transmitting CTC and participate in the process of cooperation. To check the eligibility, each RN calculates the propagation delay of two links, such as S to RN and RN to D. The RN checks the eligibility as

(2) $$\tau_{S,RN}<\tau_{S,D}\;\&\;\tau_{RN,D}<\tau_{S,D}.$$

where $\tau_{S,RN}$, $\tau_{RN,D}$ and $\tau_{S,D}$ denote the propagation delays between S and RN, S and D, and RN and D, respectively. & operation is use to to perform a logical conjunction. If RN satisfies the Equation (2), RN is eligible to participate in the process of the retransmission. After that, each RN checks its compatibility as

(3) $$Pr_{c}=1-F_{x}\left(Dis\right).$$

where $F_{x}\left(Dis\right)$ is represented by the function of the source-destination difference. Here, Dis represents as the minimum difference in propagation delay between two RNs ($l_{1}$ and $l_{2}$) without causing a collision at the source node. Now, $F_{x}\left(Dis\right)$ is expressed as

(4) $$F_{x}\left(Dis\right)=\int_{0}^{d_{max}}dL_{2}\int_{l_{2}-dis}^{l_{2}+dis}f_{l_{1},l_{2}}\left(l_{1},l_{2}\right)dL_{1}.$$

If $L_{1}$ and $L_{2}$ are random values and presents the propagation delay from two RNs to S, $f_{l_{1},l_{2}}\left(l_{1},l_{2}\right)$ is the joint probability density function. The maximum transmission range is represented as $d_{max}$. If we have a total of *x* RNs, the probability that *y* RN is compatible by ensuring that a sender should have at least one compatible RN.

(5) Prcy=x−1y(Prc)y(1−Prc)x−1−y.

In Equation (5), x−1 is the total number of RNs taking part in the process of cooperation. Each RN checks its compatibility by using Equations (3)–(5). The compatible RNs minimize the collision of control packets. The eligible and compatible RNs reply with a control packet, i.e., clear to cooperate (CTC). Additional information, i.e, propagation delay from RN-D is attached with CTC before transmitting to S. Note that if D is ready to accept the data packet from S, D responds with CTS.

#### 3.1.2. Reduce Control Packet Collision

**Figure 2:**
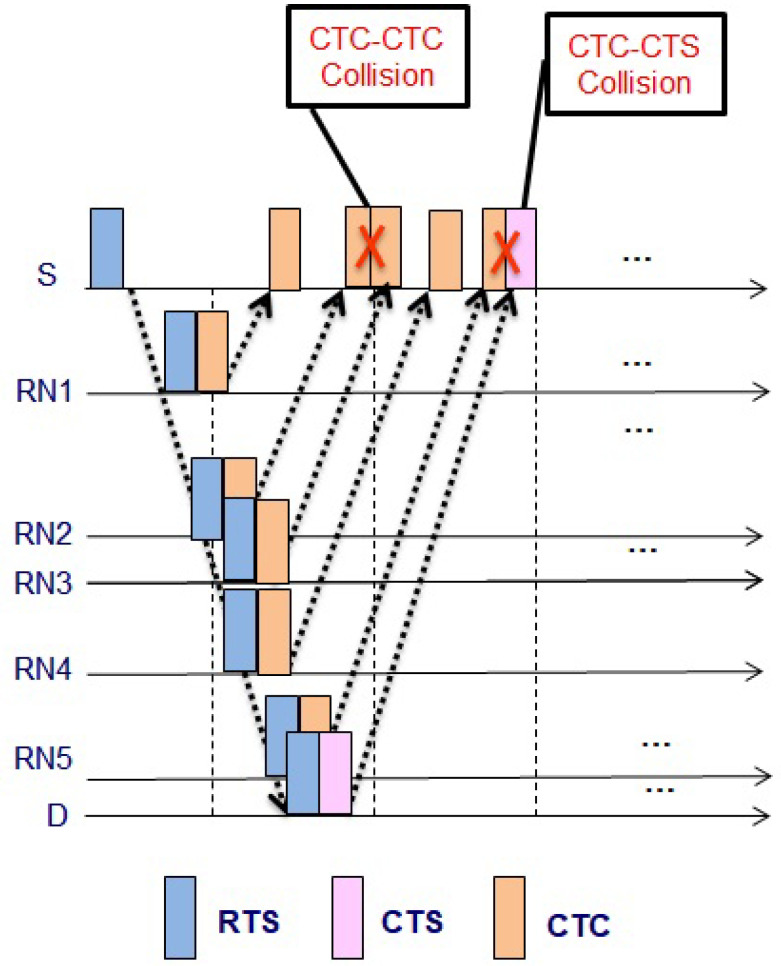
CTC-CTC and CTC-CTS collisions.

**Figure 3:**
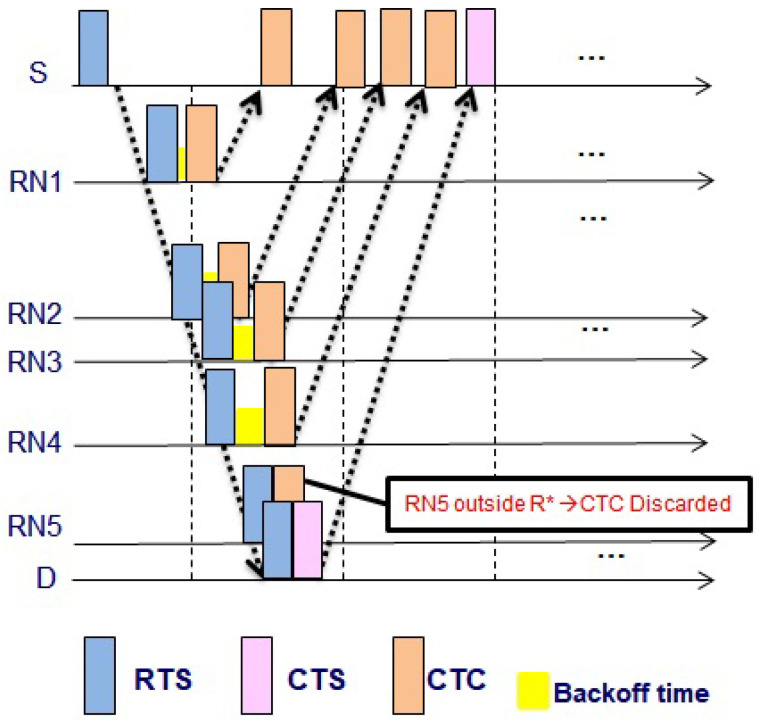
Avoid CTC-CTC collisions.

When the CTC collision from different RNs at S occurs, S becomes deprived of potential relying. Since the propagation delay is very long in the UWA channel, the collision is crucial. Even if the small parts of the packets are overlapped at S, the collision happens. Two types of collisions at S can occur, i.e., CTC-CTC and CTC-CTS collisions. CTC-CTC collision at S is caused by RNs whose distances to S are similar. In Figure 2, RN4 and RN3 have a similar distance to S, and when RN4 and RN3 reply CTC to S, the CTC-CTC collision occurs. To avoid CTC-CTC collision, RNs must send the packet with a back-off time, which is calculated from the channel gain, e.g., the RSSI of the received RTS. The back-off time can be calculated as below

(6) $$T_{backoff}=\frac{RSSI_{S,RN}}{RSSI_{low}}\psi .$$

where $RSSI_{low}$ denotes predetermined small constant value, and $\psi =T_{CTC}+\tau_{S,RN}$, and $T_{CTC}$ denotes a transmission time of the CTC packet. Since all RNs have the different RSSI values, the calculated backoff times are different and keep apart the control packets at S. Hence reduce the collision of the CTC-CTC control packets. Equation (6) reduces the collision that can be shown in Figure 3.

The CTC-CTS collision is a more crucial problem than CTC-CTC collision because if the CTC-CTS collision occurs, S is unable to listen to anything from D and the whole procedure of RTS/CTC is inadequate. In order to avoid collision among CTC-CTS, each RN first checks whether it lies in eligibility region $R^{*}$ or not by using Equation (7). The authors in [30] calculated the region ($R^{*}$) of RN, which is expressed as

(7) $$R^{*}=\left\{\begin{array}{c} 0,\;\mathrm{if}\;\frac{1}{4}d_{d}^{2}-\frac{\log2}{2*k_{d}}\leq 0\\ \sqrt{\frac{1}{4}d_{d}^{2}-\frac{\log2}{2*k_{d}}},\;\mathrm{if}\;\frac{1}{4}d_{d}^{2}-\frac{\log2}{2*k_{d}}>0 \end{array}\right.$$

where $k_{d}=c_{\phi }v_{d}^{\left(2/\phi \right)}\lambda_{d}$, $c_{\phi }=\left(2\pi /\phi \right)\Gamma \left(2/\phi \right)\Gamma \left(1-2/\phi \right)$, and ϕ donates a path loss component with $\Gamma \left(x\right)=\int_{0}^{\infty }y^{x-1}e^{-y}dy$, $v_{d}$ represents the target SNR for particular system, $d_{d}$ is the mid point of direct path between S and D, and $\lambda_{d}$ is the Poisson Point process (PPP) distribution. If RN lies within the region $R^{*}$, its transmits CTC by following the procedure given in Section 3.1 and Section 3.2. Otherwise, if the RNs lie near to D, it does not transmit CTC and CTC-CTS collision is successfully avoided.

#### 3.1.3. Best Relay Node Selection

For the selection of best RN, S firstly need to check whether the required number of RNs are capable for the retransmission. As, each RN knows the propagation delay from RNs to D ($\tau_{RN,D}$) and sends $\tau_{RN,D}$ to S along with CTC control packet. After receiving CTC, S decodes the $\tau_{RN,D}$. Now, S generates the list of the RNs that are the candidates to play the role of cooperators. S sorts the list of RNs in the order of the smallest propagation delay from the midpoint ($(\tau_{S,RN}+\tau_{RN,D})/2$) of the direct link ($\tau_{S,D}$). The channel reservation phase completes when S generates the list of RNs and ready to transfer the data packets to D. The pseudo-code for the channel reservation phase is shown in Algorithm 1.

### 3.2. Data Transmission Phase

#### 3.2.1. Broadcast Information of Selected Relay Node

**Figure 4:**
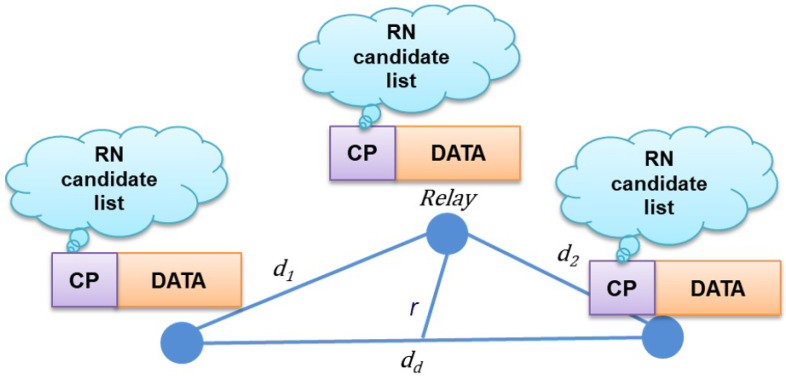
Transfer selected cooperator information.

S attaches the names of the RN candidates along with the data packet and transmits it to the D. Each RN overhears the data packets and searches whether its name is in the RN candidate list or not. When RN finds its name and the order in the list, RN saves the data packet into the buffer and wait for the retransmission request from the D. If the RN order number is two, the first NACK from D is discarded by RN. When the second NACK is received, the RN retransmit data packet. However, if the RN does not find its name on the list, it discards the data packet and starts an idle mode, e.g., sleep mode. Thus, only RNs whose names are on the list are active to listen to NACK/ACK from D. This procedure decreases the energy consumption of the RNs, whose battery powers are limited in the underwater environment. As among all RNs, only a few selected RNs buffer the data packet to take part in the process of retransmission. In Figure 4, data transmission phase of the proposed method is shown. The S broadcasts the CP and data packet, and CP contains the RN candidate list.
**Algorithm 1** Initial Channel Reservation Phase**1 Operation performed at S****2** S transmit RTS**3** CTCs received**4** Sort RN –> mini. distance to midpoint**5** Create list of RNs**6Operation performed at RNs**
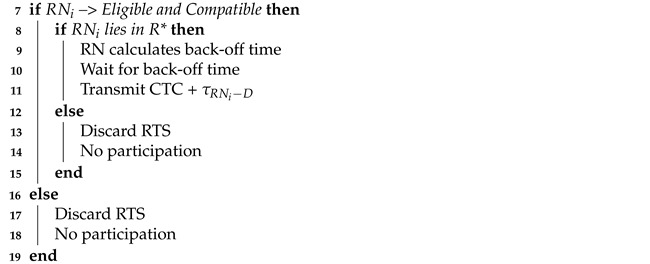
**20Operation performed at D****21** Transmit CTS

#### 3.2.2. Data Retransmission

If D does not successfully decode the information of the received data transmitted from the S, D sends NACK, which is received not only by S but also by RNs. The first priority RN listened to NACK from D and transmits the buffered data packet to D. When the D receives and successfully decode the data packet, it broadcasts ACK. After receiving an ACK, RN in candidates list discards data packet, and all RNs and S goes to the sleep mode. If D fails to decode again, it replies with a NACK and the second priority RN retransmits the data packet to D. Thus, D receives the data packet transmitted a second time by the second priority RN. S waits until the whole process of retransmissions from the RN is completed. In Equation (8), total waiting time ($T_{waiting}$) at S is formulated as

(8) $$T_{waiting}=2*\left(\tau_{S,D}+T_{CTC}\right).$$

**Figure 5:**
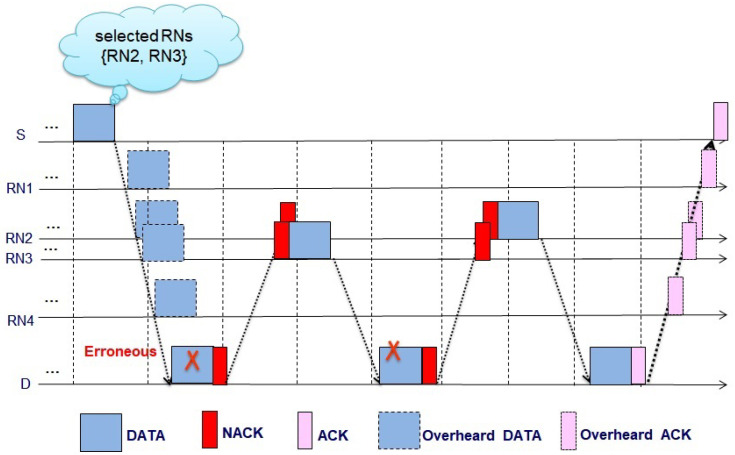
Data packet transfer in EBRN.

This overall cooperative communication scenario is displayed in Figure 5. Here, we assume that RN2 and RN3 are in the RN candidate list and successfully decodes the data packet from S. When D fail to decode from the data packet from S and sends NACK, RN2 retransmits the data packet to D. In this case, D and RN3 listen to the data packet but RN3 discards the data packet since RN3 does not want to decode the data in this phase but waits for the second retransmission. If the data packets from RN2 are not successfully decoded and the second NACK is transmitted from D, RN3 transmits the data packets to D. Note that the RN2 does not respond to the second NACK as the data packet from RN2 already fails during retransmission. This retransmission procedure is performed up to all the RN candidates. The pseudocode of the retransmission phase is shown in Algorithm 2.
**Algorithm 2** Data Transmission Phase**1 Operation performed at S****2** S transmit Data packet + list of RNs (RN${}_{i}$)**3 Operation performed at RNs****4** RN decode Data packet + list of RNs
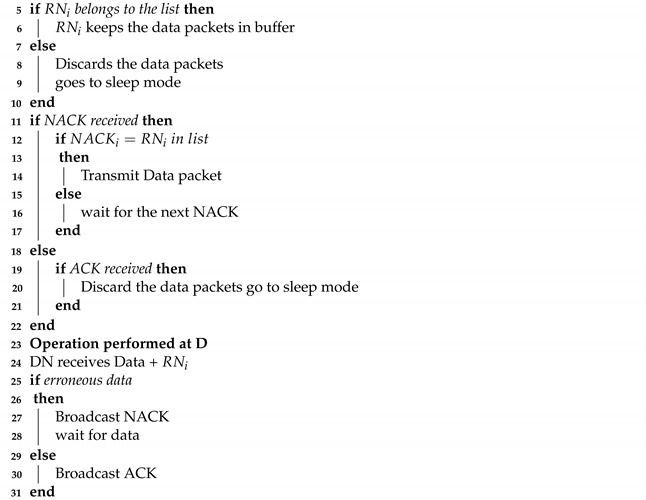


## 4. System Level Simulation Results

### 4.1. UWA Network Model

The computer simulations compare the performance of the proposed EBRN with that of the conventional UCMAC [28] and the SPF [29]. The EBRN selects RN around the middle of direct path (S-D) that ensures the reliability of both links, i.e., S-RN and RN-D, thus outperform UCMAC. Moreover, EBRN utilizes the propagation delay information that is more accurate than time which gives edge in selecting the best RNs and gives better performance than SPF. We evaluate the performance of EBRN by comparing the throughput, latency, energy efficiency, packet loss rate (LR) and packet delivery ratio (PDR). The performance metrics for our scenario can be defined as

*Throughput*: Total number of $P_{k}$ data bits (data packets) received successfully by the D per second (*Time*).

(9) $$Throughput=\frac{1}{Time}\sum_{k}P_{k}.$$

*Latency:* The average total time in seconds measured from the delays $DL_{trans}$ caused due to data packet start transmitting at the S, propagates with a time delay $DL_{S-D}$ in the channel, and successfully received at the D after retransmission delay $DL_{RN_{i}-D}$ for a particular $RN_{i}$. The formula is given as

(10) $$Latency=\left\{\begin{array}{c} DL_{S-D}+DL_{trans},No\;retransmission\\ DL_{RN_{i}-D}+DL_{S-D}+DL_{trans},Retransmission \end{array}\right.$$

*Energy Efficiency:* The total number of bits *M* in a *k* number of data packet (DP) which is first averaged and then divided by the energy consumed $E_{x}$ in joule *J*.

(11) $$E_{e}ff=\frac{1}{E_{x}}\sum_{k\in DP}M_{k}.$$

*Packet loss Rate:* The total number of data packets lost $N_{loss-pkts}$ in a system until the successful reception at the D.

(12) $$PKT_{loss}=\sum N_{loss-pkts}.$$

*Packet Delivery Ratio:* The total number of data successfully received $pkt_{suc}$ at the D divided by the total number of packets generated $pkt_{gen}$.

(13) $$PDR=\frac{\sum pkt_{suc}}{\sum pkt_{gen}}.$$

**Figure 6:**
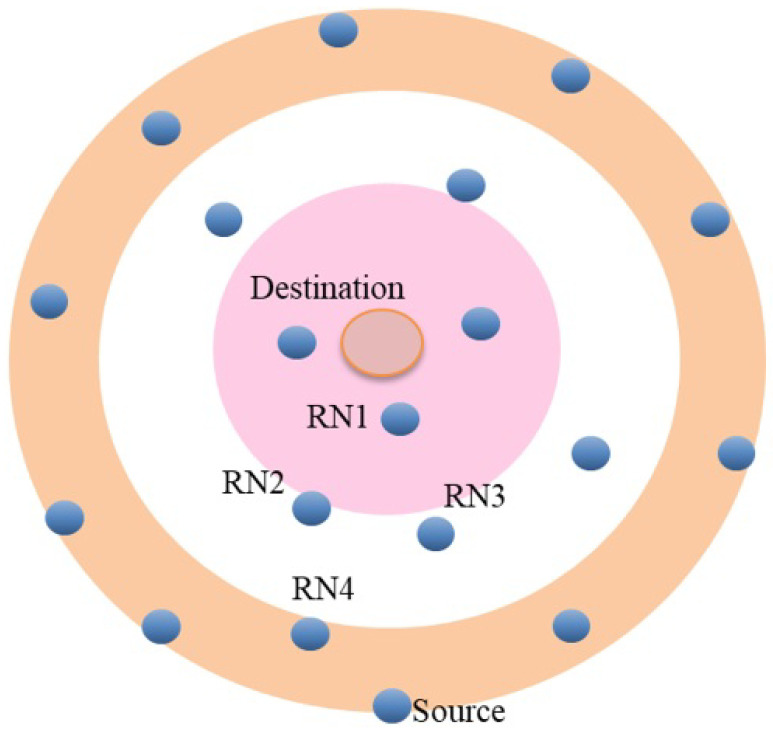
UWA Layout.

The UWA communication network consists of one S and one D and 18 static RNs that are randomly located as in Figure 6.

S generated the data packets followed by PPP with packet generation rate λ=0.001. The empirical UWA model was made from a point of W. Sea of S. Korea, which was 4.2 km apart from Sinzindo. Assume that one or more than one-bit errors in the data packet were considered as the error, while the control packets were error-free. This assumption was reasonable because the control packets are robustly encoded for low errors in the general communication standard. The total time of the successful data reception was measured by the processing time at the RNs and the propagation times from S to RN and RN to D. The propagation time was estimated by using the distance of S considering the sound speed from S to D. The propagation speed in the underwater was set as 1500 m/s. System parameters are given in Table 1.

### 4.2. Results and Discussions

**Figure 7:**
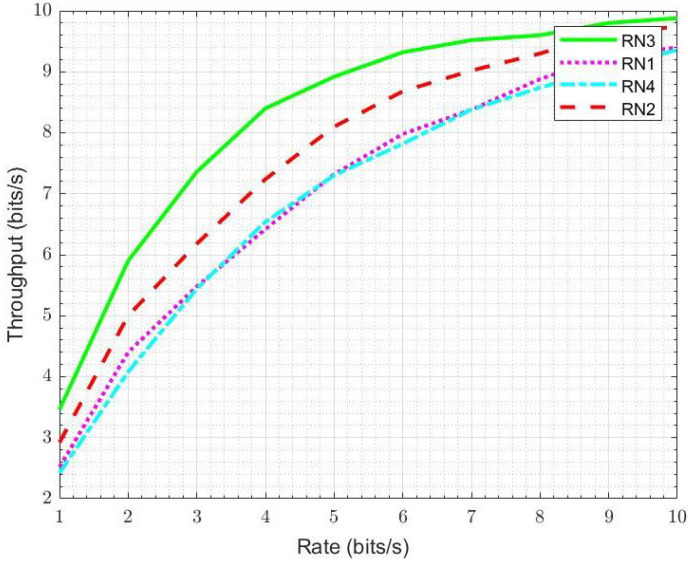
Throughput of four RNs.

**Figure 8:**
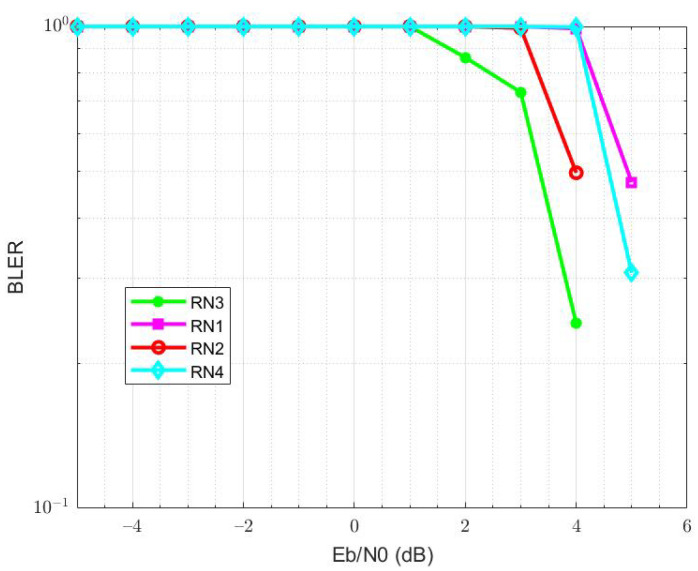
BLER of four RNs.

We compare the throughput of four RNs, located between S and D, and calculate the throughput of RNs by adding the quality of two links, i.e., S-RN and RN-D. In the proposed EBRN, RN3 belongs to the list of cooperating RNs that showed the maximum throughput among the four RNs shown in Figure 7. From the figure, it can be seen that at the rate of 5 bits/s, RN1 and RN4 throughput is around 7.2 bits/s, RN2 throughput is 8 bits/s, RN3 throughput is around 8.9 bits/s. In Figure 8, we can see that the nearest RN to D is RN1 and RN4 is closest to S. RN1 and RN4 need more power to reduce errors. We calculate BLER by considering two links, i.e., $S-RN_{i}$ and $RN_{i}-D$. For RN1, S-RN1 is very long, which causes more error in a received signal and require more SNR to overcome the errors. Similar is the case with R4, whose link R-D has many errors. RN3 and RN2 show good performance but RN3 that lies nearer to mid-point of direct link has minimum BLER.

**Figure 9:**
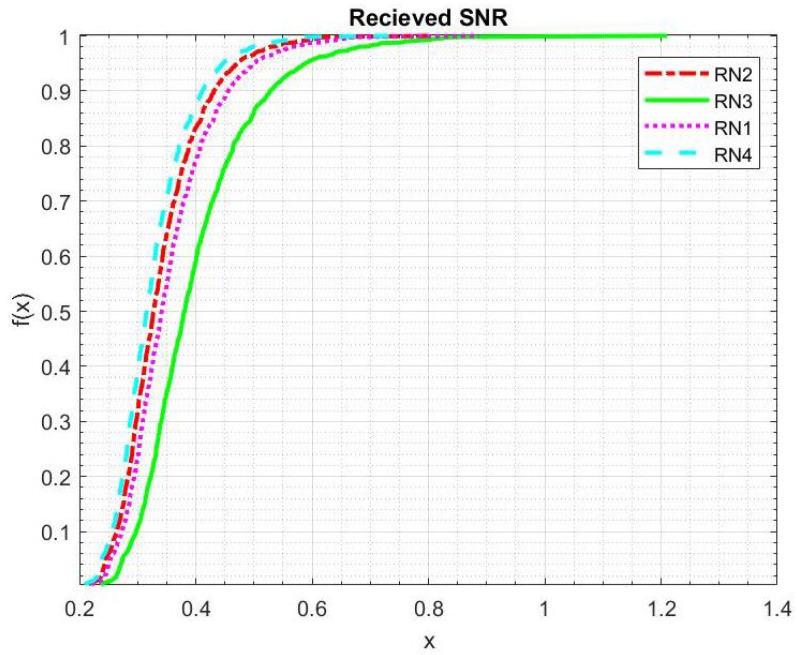
SNR of four RNs.

**Figure 10:**
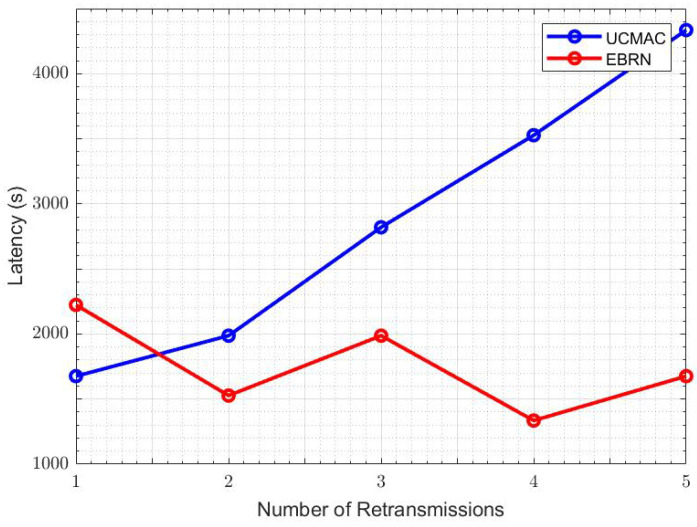
Number of retransmissions vs. Latency.

Figure 9 shows the overall SNR of the channels related to four RNs. Here, both links, S to RN and RN to D, have been considered. It shows that the SNR of RN3 (top priority in the candidate list) in EBRN has better SNR performance than other RNs. Figure 10 demonstrates that the latency of UCMAC increases with retransmissions, while in EBRN, mostly selected RNs locates around mid-point, so latency is consistent. Latency in the underwater environment is strongly affected by the number of retransmissions. Due to the long propagation delay problem in the underwater channel, the author in [28] assumed that RN nearest to the D is best for the retransmission procedure. The RN that is nearest to D is furthest from the S. In this case, S to RN link is almost similar to S to D link. The procedure follows as D asks the nearest one for retransmission, and if the nearest one RN also has an erroneous data packet, D will ask to the second closest and so on. If data received at RNs is erroneous, NACK transmission for that RN is just a waste of time and energy. In EBRN, the RN is nearest to the midpoint between S and D direct link. In this way, S to RN and RN to D has almost similar propagation delay. RN will have more chances to have error-free data packets in both connections. The path between S and selected RN is small, relative to the RN that lies nearest to D. So it is ensured that the data received at chosen RN is error-free, and D has to receive the data packet from RNs by sending control packets. There is a high possibility to have a one-time transmission of NACK in EBRN than in UCMAC. Overall performance of EBRN outperforms the UCMAC and SPF hence proving that this scheme is a well-coordinated cooperation scheme.

**Figure 11:**
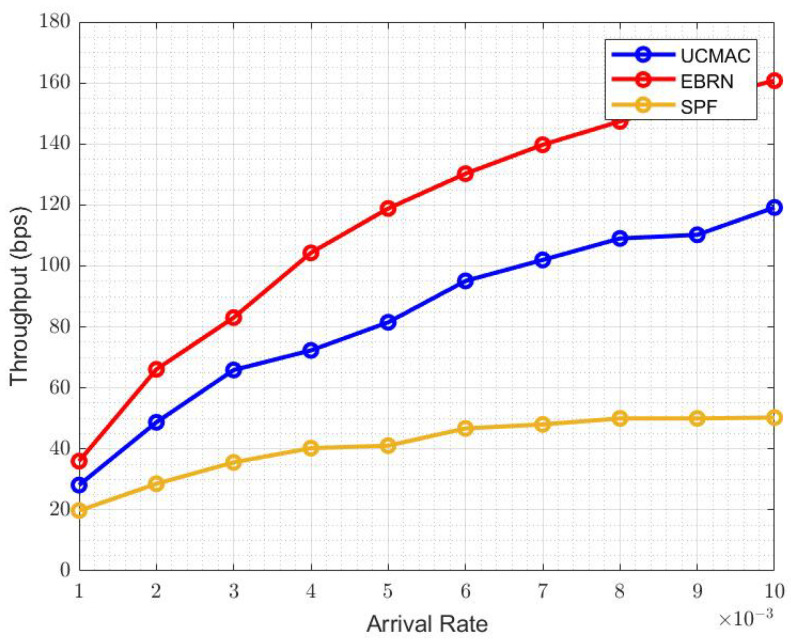
System Throughput.

**Figure 12:**
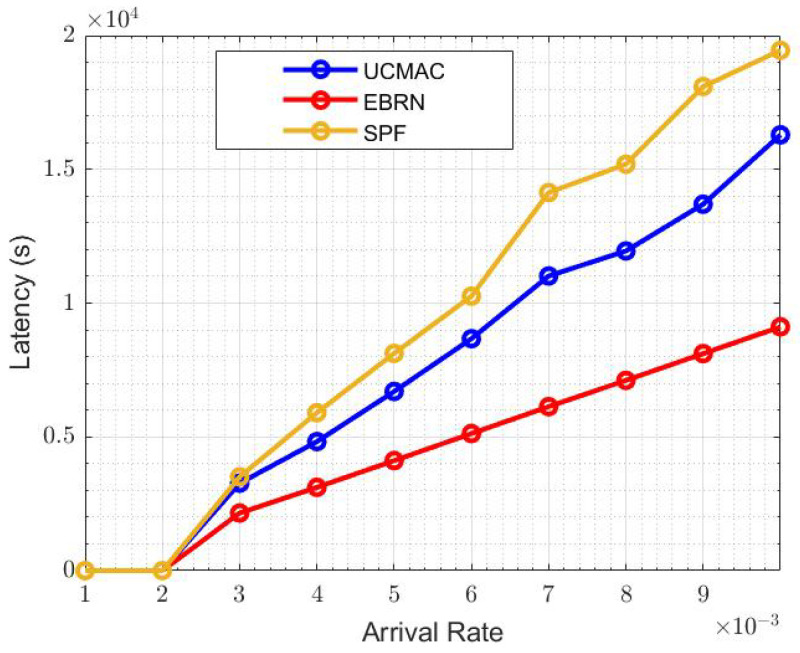
Latency.

Figure 11 shows the overall system throughput of EBRN, UCMAC and SPF. EBRN throughput is highest which is due to the less time interval between data generation and reception than in UCMAC and SPF. It means that S spends more time idle while waiting for the successful reception of the data packet. When the arrival rate is $6\times 10^{-3}$, the throughput of UCMAC and SPF is around 96 and 82 bits per second, respectively, while EBRN is almost 130 bits per second. In EBRN, data packets are received by D in less time than in UCMAC and SPF. As the RNs lie around the midpoint, that has almost similar links from S-RN and RN-D. That’s why the latency in EBRN is less than UCMAC and SPF. In EBRN, there is less number of retransmissions by the D until it receives the data packet successfully. The overall latency is shown in Figure 12. In UCMAC, mostly first retransmission is unsuccessful, so D asks for a second retransmission that increases latency. In SPF, RN near to S is far from D, and the travel time of NACK is long, which increases the overall latency. At the arrival rate of $6\times 10^{-3}$, the latency of UCMAC and SPF is $0.85\times 10^{4}$ and $1\times 10^{4}$, respectively. While EBRN latency is around $0.55\times 10^{4}$, which shows that the Latency of UCMAC and SPF is higher than EBRN. Latency includes the traveling time from D to RN and then RN to D. If this whole procedure repeats more than one time, it will definitely increase the overall latency. Generally, the RN near to S can have an error-free data packet for the S-RN link. Similarly, there are fewer chances to have data with error from the RN that is nearest to D. Hence, in both prementioned cases, one path is good and other is worst. As the data packet is large in size (long in time), only one-bit error leads to an error in the full data packet. Long-distance between nodes decreases the power of the signal that causes error in data packets. EBRN is more energy-efficient than UCMAC and SPF because there are more losses in the packet packets. Total energy consumed is calculated when each data packet transmitted from the S or RN to the D. In EBRN, a small number of data packets are dropped than in UCMAC and SPF. RN nearest to D has very few chances to have erroneous data, but the energy consumption is very high when a D contacts the 2nd nearest RN and so on. Hence, RN closest to D is furthest from S that causes more errors in the data packet. In SPF, RN that has a small ToA to the S is furthest from D. It means that the RN-D link is long and more sensitive to the intense underwater channel.

**Figure 13:**
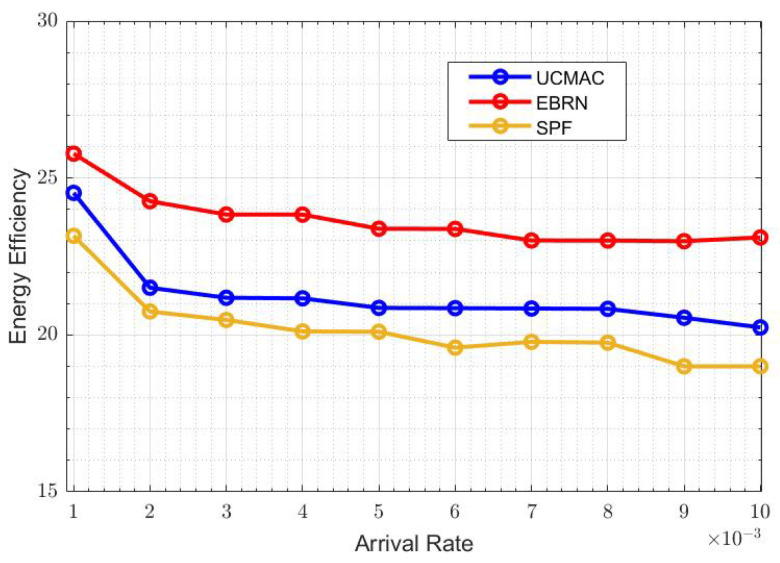
Energy Efficiency.

**Figure 14:**
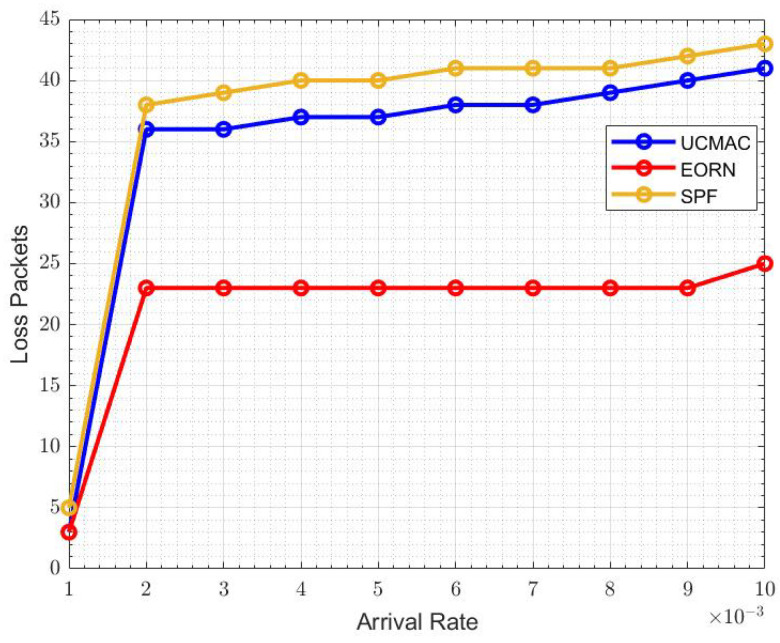
LR.

**Figure 15:**
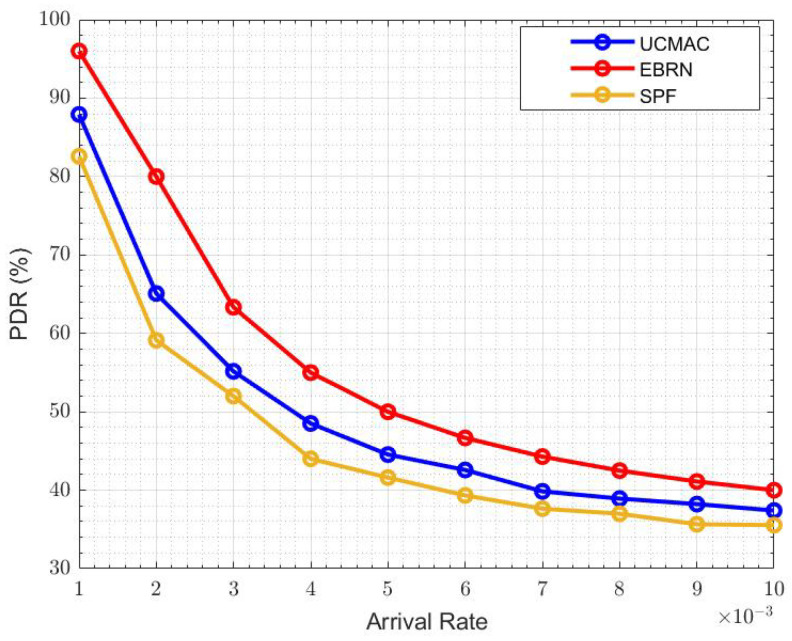
PDR.

Figure 13 sows the overall energy efficiency represents. The energy consumed when the data packet is generated and transmitted at the sensor node. If there are more erroneous data packets, there will be more energy consumption. When the arrival rate is $6\times 10^{-3}$, EBRN is more energy-efficient, and its efficiency is around 24, while in UCMAC, and SPF efficiency becomes lower, i.e., 21 and 19, respectively. In Figure 14, we can see the fewer packets are lost in EBRN than in UCMAC and SPF as in EBRN error-free data receives at one retransmission. At the arrival rate of $6\times 10^{-3}$, the LR for UCMAC and SPF is around 36 and 40 packets, while in EBRN, it’s almost 24 packets. In EBRN, Collision among data packets is reduced by considering the control packet collision avoidance methods mentioned in Section 3. In EBRN, the lost data packet decreases because of the one-time retransmission at a given time. It also increases the packet delivery ratio accordingly, seen in Figure 15. At the arrival rate of $6\times 10^{-3}$, the PDR for UCMAC and SPF is around 38% and 42%, respectively. Whereas, in EBRN, PDR is almost 50%. In all the given results, EBRN outperforms UCMAC and SPF.

## 5. Conclusions

This paper proposes a cooperative RN selection rule for UWAs named EBRN. The UWA channel is error-prone and changeable. The procedure of cooperative retransmission utilized as it provides spatial diversity and improves reliability. In EBRN, the S selects the best RN by using the propagation delay information. S chooses a list of RNs based on the nearest to the mid-point of a direct link between S and D. S sends the information of selected RNs along with the data packet to the D. RNs overhear, estimate their eligibility and keep it active to receive NACK/ACK. Those RNs whose information is not available would go to sleep mode. For retransmission, S broadcast NACK. All the active RNs continuously listen and wait for their turn. Simulation results show that EBRN performance is better in terms of throughput, latency, packet delivery ratio, energy efficiency, and LR. We have compared the results with UCMAC and SPF based on underwater cooperative protocols. The proposed scheme shows better performance than UCMAC and SPF because they have large latency and more packet losses. In EBRN, we have tried to make the first link error-free, i.e., the link between S and RN. If the first link is error-free, D asks RNs lie nearest to the midpoint between S and D direct link, which decreases overall latency. The D can receive an error-free packet if the RN lies around the mid-point of a direct link. As this research focuses on single hop cooperative communication, further investigations need to be done to develop a reliable multi-hop underwater sensor network. 

## Figures and Tables

**Table 1:** System Parameters.

Parameters	Value
Grid size	1 km × 1 km
Data packet size	1 kbits
Control packet size	120 bits
Data packet duration	0.2 s
Control packet duration	0.024 s
Number of allowed RNs	4

## Data Availability

Not applicable.
